# Phellinus linteus activates Treg cells via FAK to promote M2 macrophage polarization in hepatocellular carcinoma

**DOI:** 10.1007/s00262-023-03592-3

**Published:** 2024-01-19

**Authors:** Feihua Chen, Mouchun Gong, Dengcheng Weng, Zhaoqing Jin, Guofeng Han, Ziqiang Yang, Junjun Han, Jianjiang Wang

**Affiliations:** Department of General Surgery, Hangzhou Medical College Affiliated Lin’an People’s Hospital, No. 548 Yijin Road, Jincheng Street, Hangzhou, 311300 Zhejiang China

**Keywords:** Hepatocellular carcinoma, FAK, Treg, Macrophage polarization, Phellinus linteus

## Abstract

Hepatocellular carcinoma (HCC) is the most prevalent malignant tumor worldwide. Within HCC's tumor microenvironment, focal adhesion kinase (FAK) plays a critical role. Regulatory T cells (Treg) modulate the polarization of tumor-associated macrophages , but the relationship between FAK, Treg cells, and macrophages remains underexplored. Phellinus linteus (PL) shows promise as a treatment for HCC due to its pharmacological effects. This study aimed to explore the relationship between FAK and Treg-macrophages and to assess whether PL could exert a protective effect through the FAK process in HCC. Initially, C57BL/6-FAK^−/−^ tumor-bearing mice were utilized to demonstrate that FAK stimulates HCC tumor development. High dosages (200 μM) of FAK and the FAK activator ZINC40099027 led to an increase in Treg (CD4^+^CD25^+^) cells, a decrease in M1 macrophages (F4/80^+^CD16/32^+^, IL-12, IL-2, iNOS), and an increase in M2 macrophages (F4/80^+^CD206^+^, IL-4, IL-10, Arg1, TGF-*β*1). Additionally, FAK was found to encourage cell proliferation, migration, invasion, and epithelial-mesenchymal transition while inhibiting apoptosis in HepG2 and SMMC7721 cells. These effects were mediated by the PI3K/AKT1/Janus Kinase (JAK)/ signal transducer and activator of transcription 3 (STAT3), and mitogen-activated protein kinase (p38 MAPK)/Jun N-terminal Kinase (JNK) signaling pathways. Furthermore, PL exhibited a potent antitumor effect in vivo in a dose-dependent manner, reducing FAK, Treg cells, and M2 macrophages, while increasing M1 macrophages. This effect was achieved through the inhibition of the PI3K/AKT/JAK/STAT3, and p38/JNK pathways. Overall, our findings suggest that FAK promotes HCC via Treg cells that polarize macrophages toward the M2 type through specific signaling pathways. PL, acting through FAK, could be a protective therapy against HCC.

**Supplementary Information:**

The online version contains supplementary material available at 10.1007/s00262-023-03592-3.

## Introduction

Hepatocellular carcinoma (HCC) represents a major global healthcare challenge [1]. It accounts for 45% of deaths from HCC occur in China [2]. Chemotherapy is important in cancer treatment. However, the current chemotherapy drugs cause great damage to other organs while treating cancer [3]. Therefore, it is urgent to further study the pathological mechanism, exploring new approaches and targets of HCC.

Tumor-associated macrophage (TAM) inhibits the immune function and helps tumor cells to achieve immune escape in HCC [4]. Classically activated macrophages (M1) and alternatively activated macrophages (M2) are two polarized states of macrophages, which have completely different functions [5]. While M1 macrophages release pro-inflammatory cytokines like Interleukin-12 (IL-12) to bolster T-cell proliferation and function, thereby curtailing tumor formation and growth, M2 macrophages secrete cytokines such as IL-10. These cytokines foster tumor growth, migration, and metastasis, and dampen immune responses, facilitating immune tolerance toward tumor cells [6]. Therefore, the dysregulation of macrophage polarization may be crucial in HCC.

Apart from that, in HCC patients, CD4^+^CD25^+^ regulatory T cells (Tregs) are significantly increased in tumor-infiltrating lymphocytes and peripheral blood monocytes [7]. These Treg cells inhibit the proliferation and activity of CD8^+^T cells, disabling the response of T cells [8]. Nevertheless, reducing Treg, although some patients regain the response of CD4^+ ^ cells, cannot prevent tumor-mediated immunosuppression [9], indicating that Treg suppression is not an independent way. Further studies have found that in HCC, IL-10 is produced by TAMs to promote the differentiation of Th2 cells, reducing the number of cytotoxic T lymphocytes (CTL) [10]. The increase of Treg is accompanied by the enhancement of TAM in tumor tissues [11]. However, the production of IL-10, IL-4, and IL-13 by Treg promotes the differentiation of TAM into M2 cells [12], indicating that Tregs are vital in regulating TAM polarization. Hence, exploring the mechanism of Treg regulating TAM polarization is a new way to elucidate the pathogenesis of HCC.

Focal adhesion kinase (FAK), a non-receptor tyrosine kinase, plays a pivotal role at the crossroads of several intracellular signaling pathways, influencing a range of biological processes, from tumor genesis and growth to metastasis and apoptosis [13]. An article in Cell confirmed that FAK inhibitors help the immune system against cancer [14]. FAK (Y397) activation not only induces the depletion of CD8^+^T cells in tumor microenvironment (TME) but also promotes tumor cells to secrete chemokines/cytokines, recruiting Treg cells and inhibiting the activity of effector CD8^+^T cells [14]. Many preclinical studies have revealed that FAK signaling is closely involved in the activity of TAMs and Tregs within the TME [15]. FAK inhibitor was shown to decrease immunosuppressive TAMs and Tregs, which then increased CD8^+^T cells within the tumor and enhanced CD8^+^T cell-mediated suppression of cancerous cells [15]. Taken together, FAK may be an important molecule mediating the effect of Treg on modulating TAM polarization.

Phellinus linteus (PL), a revered traditional Chinese medicinal herb, is predominantly employed in treating tumors and autoimmune conditions. Research has substantiated the anti-tumoral properties of PL, highlighting its potency in curtailing tumor cell proliferation. Chao et al. [16] in their investigation, underscored that 3,4-dihydroxybenzalactone (DBL) derived from PL inhibits the metastasis of lung carcinoma cells through the suppression of FAK. Besides, polysaccharide (PLP) isolated from PL inhibits tumor growth and metastasis by enhancing the immune functions of macrophages, dendritic cells, NK cells, T cells, and B cells [17]. This effect of PL may be caused by releasing interferon *γ* (IFN-*γ*) from Th-1 cells, and IFN-*γ* stimulates the polarization of M2 to M1 macrophages in tumor tissues [18]. These studies suggest that PL is involved in regulating the TME and has a certain potential in the treatment of HCC, but the specific signaling mechanism needs to be further elucidated.

Therefore, this study aims to explore the mechanism of FAK on macrophage polarization through Treg and expounds on the scientific connotation of PL in the treatment of HCC.

## Materials and methods

All animals were under temperature 22 ± 2 °C, humidity 50–60%, light every 12 h(h) alternating dark, wind change 15–20 times/hours. The animal research was ratified by the Ethics Committee of the Animal Center of Zhejiang Eyong Pharmaceutical Research and Development Center. Animal use license number: SYXK (Zhe) 2021-0033.

### Construction of FAK gene knockout mice

Design of FAK guide RNA (sgRNA) and synthesis of Cas9 mRNA *(*https://zlab.bio/guide-design-resources). Cas9 mRNA was obtained by in vitro transcription and Cas9 mRNA and igsf 9-sgRNA were injected into the fertilized eggs of C57BL/6 mice. After mating, identify the mice as wild-type mice C57BL/6-FAK^+/+^ and knockout homozygous mice C57BL/6-FAK^−/−^.

### Phellinus linteus (PL) preparation

PL was activated and cultured before being filtered and dried at 60 °C. Subsequently, the dried PL was pulverized into a fine powder. This powder was then weighed and subjected to a dual extraction process with water reflux. The extracted solutions were amalgamated and concentrated to a specific volume using vacuum decompression. The concentrated solution of PL was then prepared for administration via gastric gavage.

### Establishment of H22 tumor-bearing mouse model

Mouse H22 cell line (iCell-m074) was incubated in 37 °C, 5% CO_2_. H22 cells at the right density were partially injected into the abdominal cavity of mice for culture. Finally, C57BL/6-FAK^+/+^ (*n* = 12) and C57BL/6-FAK^−/−^ (*n* = 12) weighing 8–20 g with 6–8 weeks mice were injected with H22 cells 0.2 mL at a density of 1.0 × 10^7^ /mL into the left forelimb subcutaneously.

BalB/c male mice (*n* = 10 in each group) were divided into the normal control (NC) group, model group, cyclophosphamide (CTX) (30 mg/kg, as a positive control), PL-L (100 mg/kg), PL-M (200 mg/kg) and PL-H (400 mg/kg) groups. The dosage of the PL referred to the previous work [19]. All BalB/c mice were injected with H22 cells except the NC group. 24 h later, mice in each group were given the corresponding drug intragastrically, and the NC group and model group were given normal saline 10 mL/kg, for 12 days.

### Sample collection

Visible tumor nodules appeared approximately 48 h later. Every two days for the following 12 days, the tumor volume was measured, and its growth was charted on a curve. At the end of the 12 days, mice were anesthetized using isoflurane inhalation. Blood samples were then drawn from their eyeballs, and the tumors were excised, photographed, and weighed. Moreover, these tumor tissues were procured for further analysis, including staining and Western blotting.

### *Isolation of CD4*^+^*CD25*^+^*Treg cells and macrophages*

Healthy C57BL/6 mice were used to isolate the Treg cells and macrophages. CD4^+^T cells were from spleen tissue by mouse CD4^+^T cell magnetic bead isolation kit. CD4^+^CD25^+^Treg cells were positively selected by adding PE-labeled anti-mouse CD25 monoclonal antibody and magnetic beads labeled anti-PE.

The abdominal fluid was collected, and after being centrifuged, cells were collected as macrophages.

### *Establishment of the CD4*^+^*CD25*^+^*Treg cell-macrophage co-culture system*

CD4^+^CD25^+^Treg cells and macrophages were incubated at 37 °C, 5% CO_2._ According to the study [20], CD4^+^CD25^+^Treg cells were inoculated into the upper compartment of a 6-well plate. Macrophages were inoculated into 6-well plates containing DMEM medium for culture until all cells were adherent to the wall.

### Cell groups

*Grouping 1* Different concentrations of FAK recombinant protein (0, 50, 200 μM), FAK (200 μM) + 10 nM ZINC40099027 (FAK activator) and FAK (200 μM) + 10 nM PF-573228 (FAK inhibitor) were added to Treg cell-macrophage co-culture system for 24 h.

*Grouping 2* The macrophage cells with or without the FAK treatment were co-cultured with 200 μM FAK for 24 h.

*Grouping 3*: The PI3K inhibitor (LY294002, 10 μM) and P38 inhibitor (SB203580, 10 μM) were preadded to the Treg cell-macrophage co-culture system for 30 min. The groups were divided into “co-culture, co-culture + FAK (200 μM), co-culture + FAK (200 μM) + PI3K inhibitor, co-culture + FAK (200 μM) + P38 inhibitor” groups.

All macrophages in groups were collected for subsequent experiments.

HCC cells (HepG2 and SMMC7721) were supplied by the Icell company, China (iCell-h092, iCell-h197) and cultured in a 37 °C 5% CO_2_. The collected macrophages above were used to added into the HepG2 and SMMC7721 cells. Different concentrations of FAK treatment were the same as above for 24 h, after that the cells were collected.

### Hematoxylin–eosin (H&E) staining

The tumor issues were fixed, dehydrated by gradient ethanol and xylene, then immersed in wax. Then, the tissues were cut into 5 μm slices, dewaxed and hydrated by xylene and gradient ethanol, and stained with HE staining (SERVICEBIO, G1005). At last, ethanol with low to high concentrations was added to dehydrate. Vitrification by xylene and the slices were sealed.

### TUNEL staining

Deparaffinized tumor tissue sections were incubated with proteinase K (G1205, Servicebio) in a humidified chamber for 15 min, and sections were then incubated with 3% H_2_O_2_ and terminal deoxynucleotidyl transferase (TdT) (G1501, Servicebio) labeling buffer at 37 °C for 1 h. Later, stained with 4′,6-diamidino-2-phenylindole (DAPI).

### Flow cytometry (FCM)

The cells were appropriately treated with Annexin V-FITC and propidium iodide (BD, 556,547). The apoptosis rate was detected by FCM (BD Calibur). The tumor cells were lysed and were incubated with anti-CD16/32, anti-F4/80, and anti-CD206 antibodies Anti-Mouse APC (Clone:17A2) (Liankebio, AM003E0205), Anti-Mouse CD4, FITC (Clone:GK1.5) (Liankebio, AM00401), PE Cy7 (Rat anti-mouse CD25, 552,880), PC-R700(Rat anti-Mouse F4/80, 565,787), V421(Rat anti-Mouse CD16/CD32, 562,896), PE (Rat anti-Mouse CD206, 568,273) at 4 °C, washed and resuspended. The data was analyzed by Flow jo software.

### Enzyme-linked immunosorbent assay (ELISA) measurement

The serum levels of IFN-*γ* (MM-0182M1), Interleukin-2 (IL-2) (MM-0701M1), Vascular endothelial growth factors (VEGF) (MM-0128M1), IL-4(MM-0165M1), IL-10 (MM-0176M1) IL-12 (MM-44769M1) tumor necrosis factor (TNF-*α*) (MM-0132M1), were tested with ELISA kits from Meimian, following the manufacturer’s instructions.

### Immunohistochemistry (IHC) assay

The sections were dewaxed with xylene, then rehydrated, and antigen repair solution was added, then the sections were blocked, and incubated overnight with CD31(ab182981, GR6568424-1), Ki67 (ab15580, GR2982445-1), Proliferating Cell Nuclear Antigen (PCNA) (ab29, GR2569874-2), F4/80 (ab111101, GR2684642-2), Mannose Receptor (CD206) (ab64693, GR3514158-2) Liver Arginase (Arg 1) (ab96183, GR2918764-2) inducible nitric oxide synthase (iNOS) (ab283655, GR2745681-1) CD16/32 (ab228971, GR5646411-1) Goat Anti-Rabbit IgG H&L (ab6721, GR4885158-2) all from Abcam at 4 °C. the corresponding secondary antibody of HRP was incubated. DAB (SERVICEBIO, G1212) was added.

### Quantitative real-time PCR (qRT-PCR)

The RNA was obtained by Trizol (Sangon Biotech, B511311) extraction and transcribed into cDNA with a reverse transcription kit (Jiangsu Cowin Biotech CW2569). Primers, DEPC, cDNA, and SYBR Green (Takara, RR820A) were added to prepare the corresponding system for amplification products in the PCR instrument. The sequences of the primer are listed below in Table 1.

**Table 1:** Primer sequence

Gene	5′-forward primer-3′	5′-reverse primer-3′
Mouse IL-10	CTTACTGACTGGCATGAGGATCA	GCAGCTCTAGGAGCATGTGG
Mouse TGF-*β*1	GTCCAACATGATCGTGCGCT	CTTTAATAGCCCGCAGGTGG
Mouse Arg-1	CTCCAAGCCAAAGTCCTTAGAG	GGAGCTGTCATTAGGGACATCA
Mouse iNOS	TTCACGACACCCTTCACCACAA	CCATCCTCCTGCCCACTTCCTC
Mouse GAPDH	TCAACGGCACAGTCAAGG	TGAGCCCTTCCACGATG

### Western blot

The protein was quantified. After being separated by electrophoresis, the protein was transferred to the PVDF membrane. Then it was blocked and incubated with antibodies as Table 2. After incubation at 4 °C overnight, it was incubated with secondary antibodies. Finally, blots were captured in an ECL luminescence imager.

**Table 2:** Antibody information

Antibody	Company	No.	Dilution rate
IL-10 antibody	Affinity	DF6894	1:1000
TGF-*β*1 antibody	Affinity	BF8012	1:1000
Arg-1 antibody	Affinity	DF6657	1:1000
iNOS antibody	Affinity	AF0199	1:2000
PI3K antibody	Affinity	AF6241	1:1000
p-PI3K antibody	Affinity	AF3242	1:1000
AKT antibody	Affinity	AF6261	1:1000
p-AKT antibody	Affinity	AF0016	1:1000
mTOR antibody	Affinity	AF6308	1:1000
p-mTOR antibody	Affinity	AF3308	1:1000
JAK2 antibody	Affinity	AF6022	1:1000
STAT3 antibody	Affinity	AF6294	1:1000
p-STAT3 antibody	Affinity	AF3293	1:1000
p38 antibody	Affinity	AF4001	1:1000
p-p38 antibody	Affinity	AF6456	1:1000
ERK antibody	Affinity	BF8044	1:1000
p-ERK antibody	Affinity	AF1015	1:1000
JNK antibody	Affinity	AF6318	1:1000
p-JNK antibody	Affinity	AF3318	1:1000
E-cadherin Antibody	Affinity	AF0131	1:1000
N-cadherin Antibody	Affinity	AF5239	1:1000
Vimentin antibody	Affinity	BF8006	1:1000
Bax antibody	Affinity	AF0120	1:1000
Bcl-2 antibody	Affinity	AF6139	1:1000
Caspase-3 Antibody	Affinity	AF6311	1:1000
FAK antibody	Affinity	AF6397	1:1000
p-FAK antibody	Affinity	AF3398	1:1000
Anti-rabbit IgG, HRP-linked antibody	CST	7074	1:6000
Anti-mouse IgG, HRP-linked antibody	CST	7076	1:6000
*β*-actin antibody	Affinity	AF7018	1:10,000

### Immunofluorescence assay

Cells and the tumor sections were fixed. After blocking, they were incubated with iNOS (ab178945), Liver Arginase (ab96183), Vimentin (ab92547), E-Cadherin (AF013172), N-Cadherin(ab18203), GAR IgG H&L (Alexa Fluor® 488) (ab150077) all from Abcam, FAK(AF6397), p-FAK(AF3398) from Affinity overnight at 4 °C. After washing, they were incubated with anti-rabbit antibody.

### 3-(4,5-Dimethylthiazol-2-yl)-2,5-diphenyltetrazolium bromide (MTT) staining

HepG2 and SMCC-7721 cells were re-suspended into 96-well plates. MTT (BBI Life Sciences, E606334) was added to each well and cultured at 37 °C for 4 h. Then, the supernatant was removed and DMSO was added. The absorbance of samples at 490 nm was detected.

### 5-ethynyl-2-deoxyuridine (EdU) staining

When performing an EdU incorporation assay (Beyotime, C0078S), we seeded cells into a 24-well plate with EdU at 37 °C for 4 h, followed by fixation. Next, the cells were treated with 0.2% Triton X-100 and 0.5 mL Click. The ratio of EdU-stained cells to Hoechst 33,342-stained cells was used to evaluate cell proliferation.

### Colony formation assay

Cells were fixed and stained with crystal violet (Solarbio) for 0.5 h. The number of colonies was photographed and counted under an optical microscope (Olympus).

### Invasion and migration assay

Matrigel was diluted in serum-free DMEM high glucose medium, and Matrigel diluent was uniformly coated in a transwell chamber and incubated at 4 °C overnight. The cells were added to the upper chamber and cultured for 24 h. The Matrigel and the bottom cells of the upper chamber were wiped off with cotton swabs, fixed, and washed. After staining with 0.1% crystal violet, the number of cells invading the lower chamber was counted.

## Statistical analysis

SPSS software was used to conduct the statistical analysis (16.0, IBM, USA). One-way ANOVA was performed, and the Tukey test was utilized for further pairwise comparisons within groups. The information was presented as mean standard deviation. The level of statistical significance was set at 0.05.

## Results

### FAK promotes cell proliferation, migration, and invasion of HepG2 and SMMC7721 cells processed with the Treg-macrophage co-culture supernatant

#### *FAK*^*-/-*^* mice exerted anti-tumor effect against HCC and inhibited the M2/M1 macrophages and Treg cells*

**Fig. 1:**
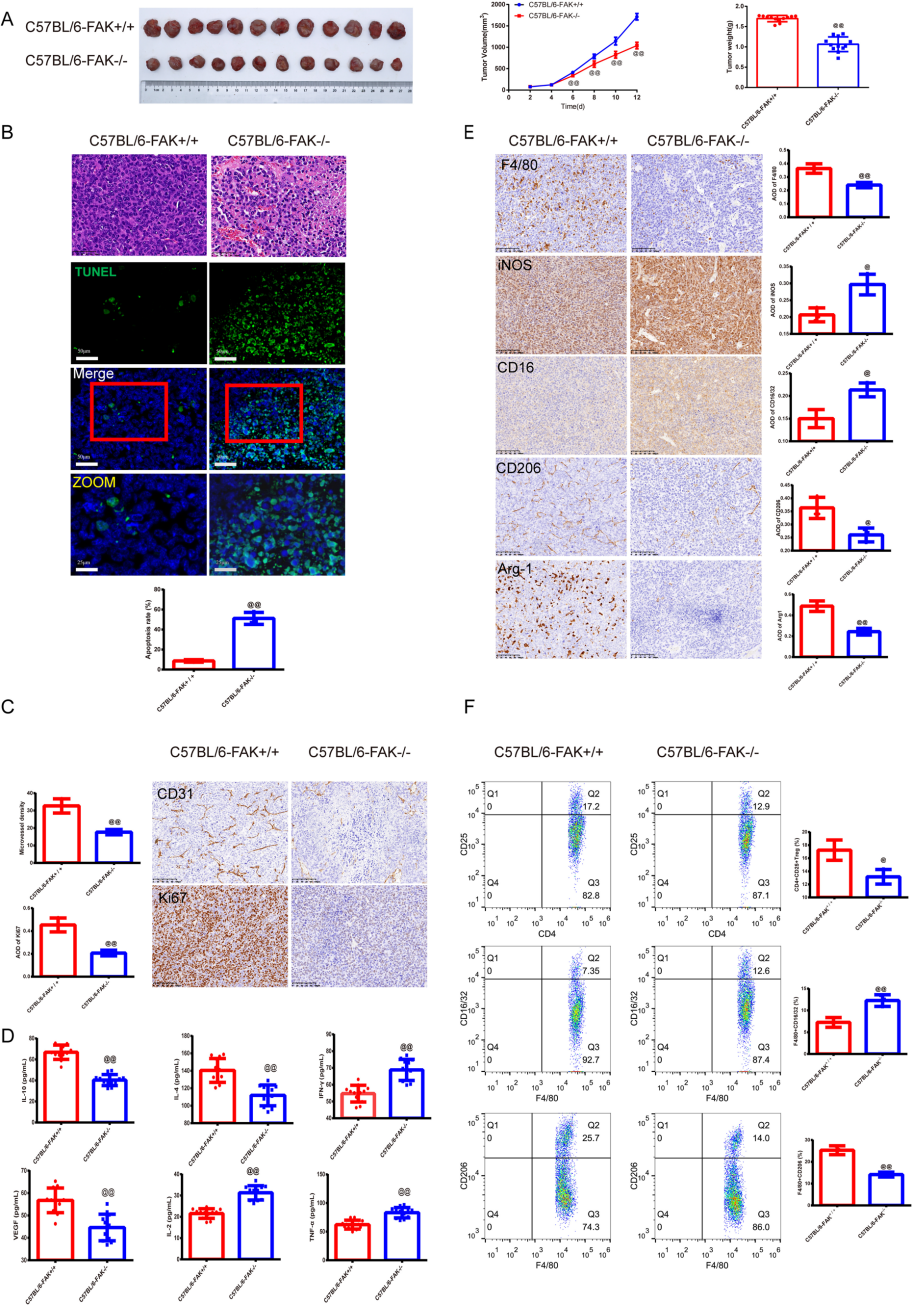
FAK^−/−^ mice exerted anti-tumor effect against HCC and inhibited the M2/M1 macrophages and Treg cells. **A** The H22 tumor volume in FAK^+/+^ and FAK^−/−^ C57BL/6 mice were recorded in 12 days. And the tumor weight was recorded on the 12th day,* n* = 12 in each group; **B** The histomorphology of the H22 tumor tissue in mice was observed by HE staining (magnification × 200, scale bar = 50 μm). TUNEL staining was used to observe the apoptosis of H22 tumor cells in mice, (magnification, × 200, scale bar = 50 μm; ZOOM, × 400, scale bar = 25 μm), and the apoptosis rate was calculated,* n* = 3 in each group; **C** The positive expressions of CD31 and Ki67 of the H22 tumor tissue in mice were examined by IHC (magnification × 200, scale bar = 50 μm), and the average of density was calculated and shown in graphs.* n* = 3 in each group; **D** The serum immune-related cytokines level of IL-10, IL-4, IFN-*γ*, VEGF, IL-2, TNF-*α* were tested by the ELISA kit,* n* = 12 in each group; **E** F4/80, iNOS, CD16, CD206, Arg 1 of the H22 tumor tissue in mice were examined by IHC in each group (magnification × 200, scale bar = 50 μm), the average of density was calculated in graphs.* n* = 3 in each group, **F** FCM was used to test the percentage of CD25^+^CD4^+^ Treg cells, F4/80^+^CD16/32^+^ M1, and F4/80^+^CD206^+^ M2 macrophages, the results were demonstrated as graphs. The abscissa axis and ordinate axis represent different amounts of protein, *n* = 3 in each group. ^@^*P* < 0.05, ^@@^*P* < 0.01 verses C57BL/6-FAK^+/+^group. *Note* *FAK* Focal adhesion kinase; *HCC* Hepatocellular carcinoma; *HE* Hematoxylin–eosin; *IHC* Immunohistochemistry; *IL-10* Interleukin-10; *IFN-γ* Interferon *γ*; *VEGF* Vascular endothelial growth factors; *TNF-α* Tumor necrosis factor; *ELISA* Enzyme-linked immunosorbent assay; *iNOS* Inducible nitric oxide synthase; *FCM* Flow cytometry; *Arg 1* Arginase 1

To delineate the influence of FAK on HCC progression, we generated FAK-deficient C57BL/6 mice (C57BL/6-FAK^−/−^) and compared tumor growth with their wild-type counterparts (C57BL/6-FAK^+/+^). In tumor growth dynamics (Fig. 1A), notably, by day 12, tumor volumes in the C57BL/6-FAK^−/−^ mice were significantly reduced compared to the C57BL/6-FAK^+/+^ mice. This reduction was further reflected in a marked decrease in tumor weight on day 12 (*P* < 0.01). In histological examination (Fig. 1B), HE staining of tumors from C57BL/6-FAK^+/+^ mice revealed pronounced cell growth, large and varied nuclei, uniform and dense cellular distribution, and an extensive capillary network. In contrast, C57BL/6-FAK^−/−^ mice exhibited tumor cells with varying degrees of vacuolation and a more relaxed tissue arrangement. Importantly, a heightened rate of apoptosis was observed in tumors from FAK-deficient mice when compared to the wild type (*P* < 0.01). In cellular markers of proliferation and angiogenesis (Fig. 1C), both CD31 and Ki67, markers for angiogenesis and proliferation, respectively, exhibited decreased expression in the C57BL/6-FAK^−/−^ mice compared to their wild-type counterparts (*P* < 0.01). In cytokine profile analysis (Fig. 1D), ELISA assays demonstrated a significant decrease in IL-10, IL-4, and VEGF levels in tumors from FAK-deficient mice. Inversely, pro-inflammatory cytokines, including IFN-*γ*, IL-2, and TNF-*α*, showed elevated expression in the C57BL/6-FAK^−/−^ group compared to the control (*P* < 0.01). In Fig. 1E, FAK knockout led to a reduction in F4/80^+^ macrophage counts. Interestingly, markers for M1-like macrophages, iNOS and CD16, were upregulated, whereas M2-like macrophage markers, CD206 and Arg-1, were downregulated in the FAK-deficient mice (*P* < 0.05 or *P* < 0.01). In FCM analysis (Fig. 1F), assessment of immune cell populations showed a decrease in the percentage of CD4^+^CD25^+^Treg cells and F4/80^+^CD206^+^ M2 macrophages in the C57BL/6-FAK^−/−^ mice. In contrast, an increased presence of F4/80^+^CD16/32^+^ M1 macrophages was observed when compared to the C57BL/6-FAK^+/+^ group (*P* < 0.05 or *P* < 0.01).

#### FAK promotes the M2 polarization of macrophages in the Treg-macrophage co-culture system

**Fig. 2:**
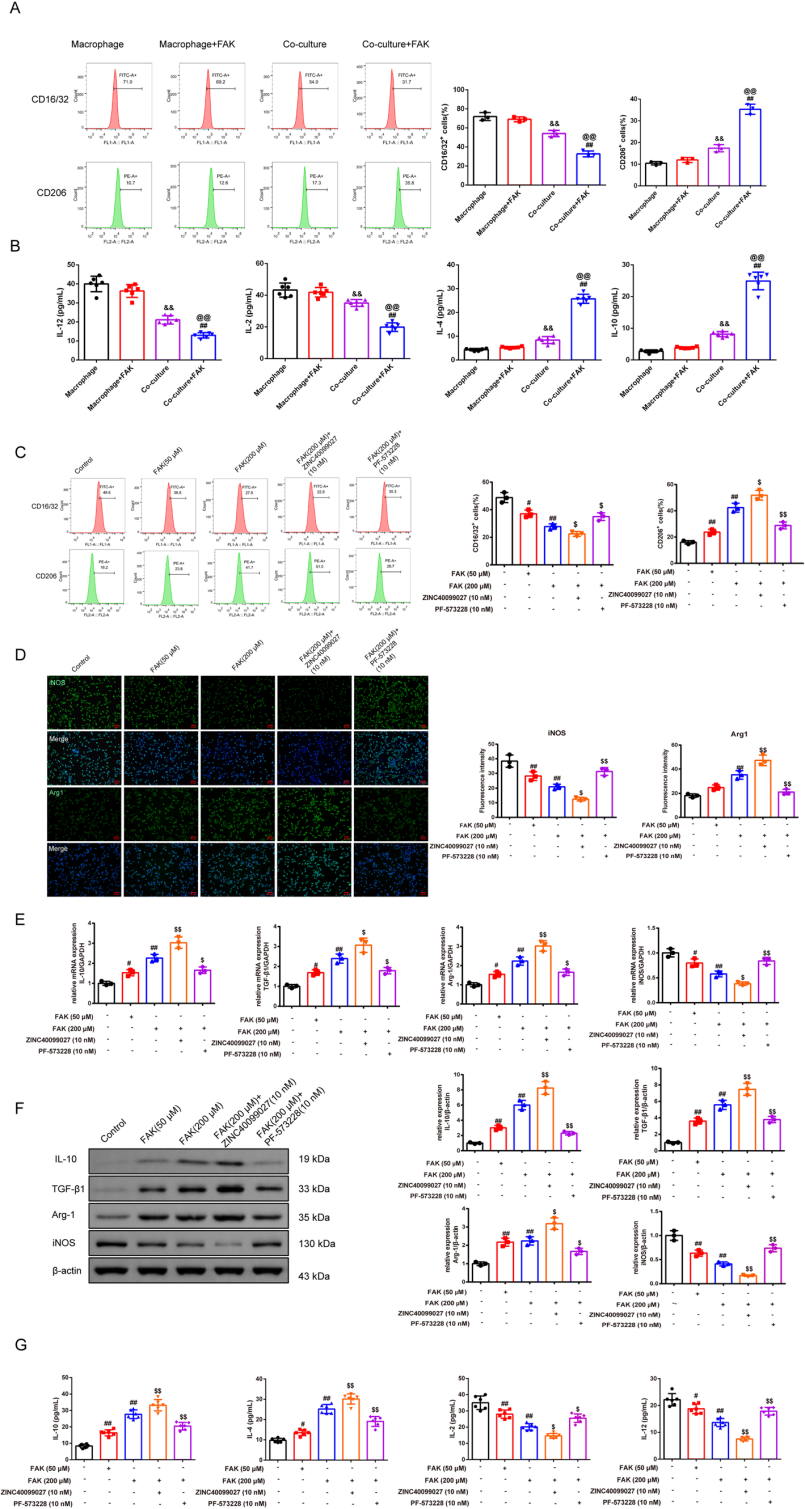
FAK promotes the M2 polarization of macrophages in the Treg-macrophage co-culture system. **A** The proportion of M1 macrophages (CD16/32) and M2 macrophages (CD206) under FAK (200 μM) inference that were co-cultured with and without Treg cells was detected by the FCM. The abscissa axis represents types of laser dyes, and the ordinate represents cell counts,* n* = 3 in each group. **B** The IL-12, IL-2, IL-4, and IL-10 contents that the macrophages released in each group were observed by the ELISA kits,* n* = 6 in each group. ^&&^*P* < 0.01 verses macrophage group, ^@@^*P* < 0.01 verses macrophage + FAK group, ^##^*P* < 0.01 verses co-culture + FAK group. **C** The proportion of M1 macrophages (CD16/32) and M2 macrophages (CD206) that were co-cultured with Treg cells was detected by the FCM after the treatment of the dosages of FAK and its activator and inhibitor in each group. The abscissa axis represents types of laser dyes, and the ordinate represents cell counts. **D** The immunofluorescence was used to test the iNOS and Arg1 protein expression in each group of macrophages that was co-cultured with Treg cells (magnification, × 200, scale bar = 50 μm), *n* = 3 in each group; **E, F** The qRT-PCR and western blot were used to test the mRNA and protein expression of the IL-10, TGF-*β*1, Arg 1, and iNOS in each group of macrophages that were co-cultured with Treg cells,* n* = 3 in each group. **G** The IL-10, IL-4, IL-2, and IL-12 contents that the macrophages released in each group were observed by the ELISA kits,* n* = 6 in each group. ^#^*P* < 0.05, ^##^*P* < 0.01 verses control group, ^$^*P* < 0.05, ^$$^*P* < 0.01 verses FAK (200 μM) group. *Note* *qRT-PCR* Quantitative real-time PCR, *TGF-β1* Transforming growth factor-*β*1

In Fig. 2A, B, under the detection of FCM and ELISA, the expression of CD16/32, iNOS, IL-12, and IL-2 (M1 macrophages), were expressed less in co-culture groups than macrophage group (*P* < 0.01). The co-culture system with the FAK (200 μM) resulted in a reduction concerning the above index compared to the co-culture group (*P* < 0.01). The CD206, IL-4, and IL-10 (M2 macrophages) got strengthened in a co-culture group than the macrophage group (*P* < 0.01). The addition of FAK strengthened the trend relative to the co-culture group (*P* < 0.01) and there was no significant difference between macrophages and macrophage + FAK groups. In Treg-macrophage co-culture system treated with the different dosages of the FAK, its activator ZINC40099027, and inhibitor PF-573228, we discovered that the polarization of macrophages has changed. In Fig. 2C, D, CD16/32, iNOS (M1 macrophages), were expressed less after the treatment of the FAK than the control group, especially with the 200 μM of FAK (*P* < 0.05 or *P* < 0.01). The ZINC40099027 with the FAK (200 μM) resulted in a further lower percentage of the M1-like macrophages compared to the FAK (200 μM) group, while the PF-573228 with the FAK (200 μM) generated the converse effect (*P* < 0.01). The CD206, Arg-1(M2 macrophages) got strengthened with FAK and activator of the FAK but decreased with the inhibitor of the FAK (*P* < 0.05). In Fig. 2E, F, with the qRT-PCR and western blot, we discovered that the mRNA and the protein expressions of the IL-10, TGF-*β*1, and Arg-1 that M2-like macrophages released, became intensified whereas the iNOS that M1-like macrophages marker was restrained after the interfere of the FAK in relative to the control group, especially with the 200 μM of FAK. The FAK activator addition had a strengthened effect compared to the FAK (200 μM) group, while the inhibitor of the FAK addition demonstrated the opposite way (*P* < 0.05 or *P* < 0.01). Consistently, in Fig. 2G, the content of the IL-10, and IL-4 were elevated while the content of the IL-2, and IL-12 were reduced with the FAK, and its activator interfered in comparison with the control group (*P* < 0.05 or *P* < 0.01).

#### FAK promotes the polarization of macrophages in the Treg-macrophage co-culture system through PI3K/AKT/JAK/STAT3 and p38/JNK/ERK signal pathway

**Fig. 3:**
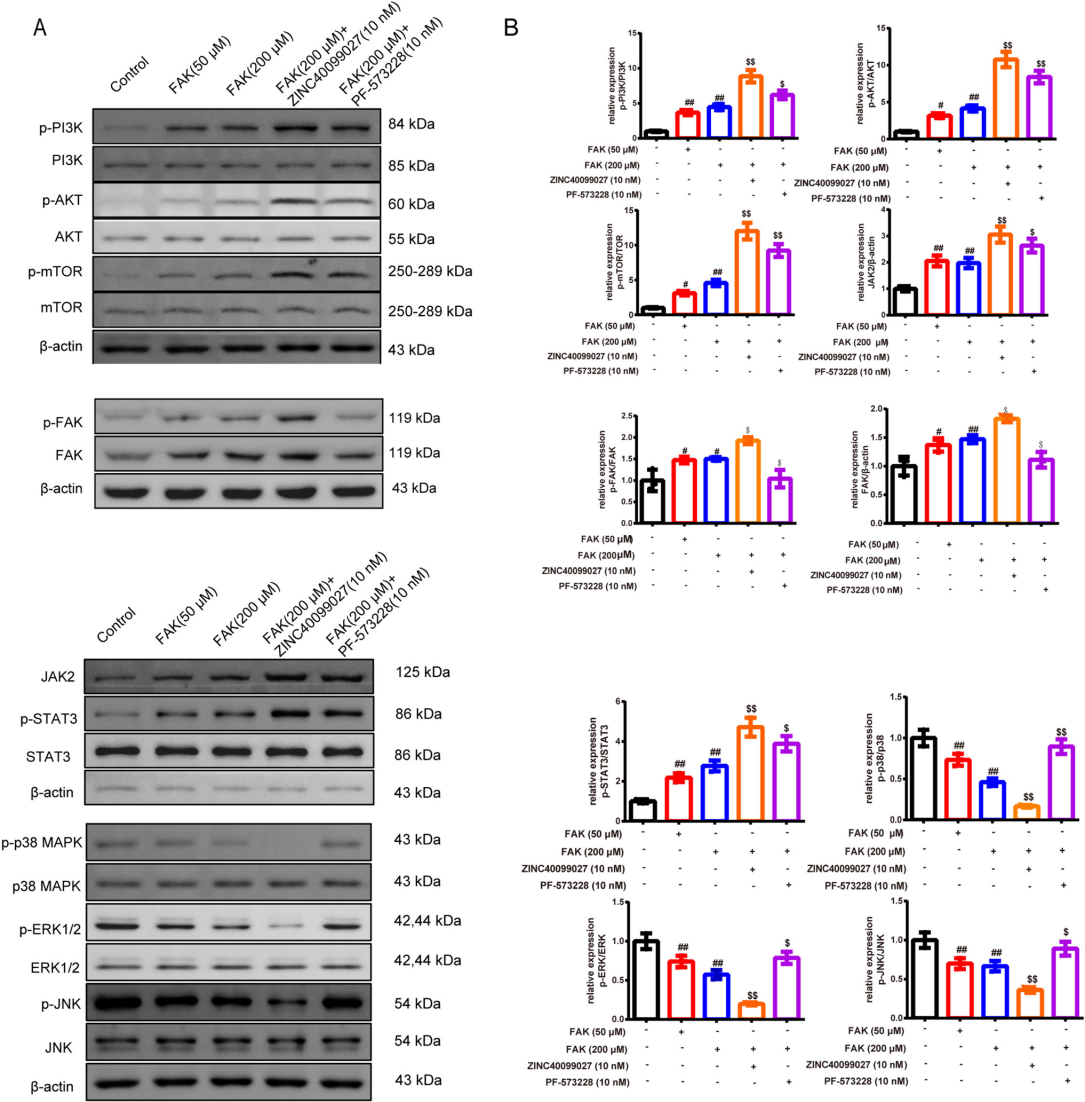
FAK promotes the polarization of macrophages in the Treg-macrophage co-culture system through PI3K/AKT/JAK/STAT3 and p38/JNK/ERK signal pathway **A, B** The expression of the PI3K, p-PI3K, AKT, p-AKT, p-FAK, FAK, JAK2, STAT3, p-STAT3 and p38, p-p38/JNK, p-JNK/ERK1/2, p-ERK1/2 in Treg-macrophage co-culture system were observed by the western blot, *n* = 3 in each group. ^#^*P* < 0.05, ^##^*P* < 0.01 verses control group, ^$^*P* < 0.05, ^$$^*P* < 0.01 verses FAK (200 μM) group. *Note* *PI3K* Phosphatidylinositide 3-kinases; *JAK* Janus kinase; *STAT3* Signal transducer and activator of transcription 3; p38 *MAPK* Mitogen-activated protein kinases; *JNK* Jun N-terminal Kinase

**Fig. 4:**
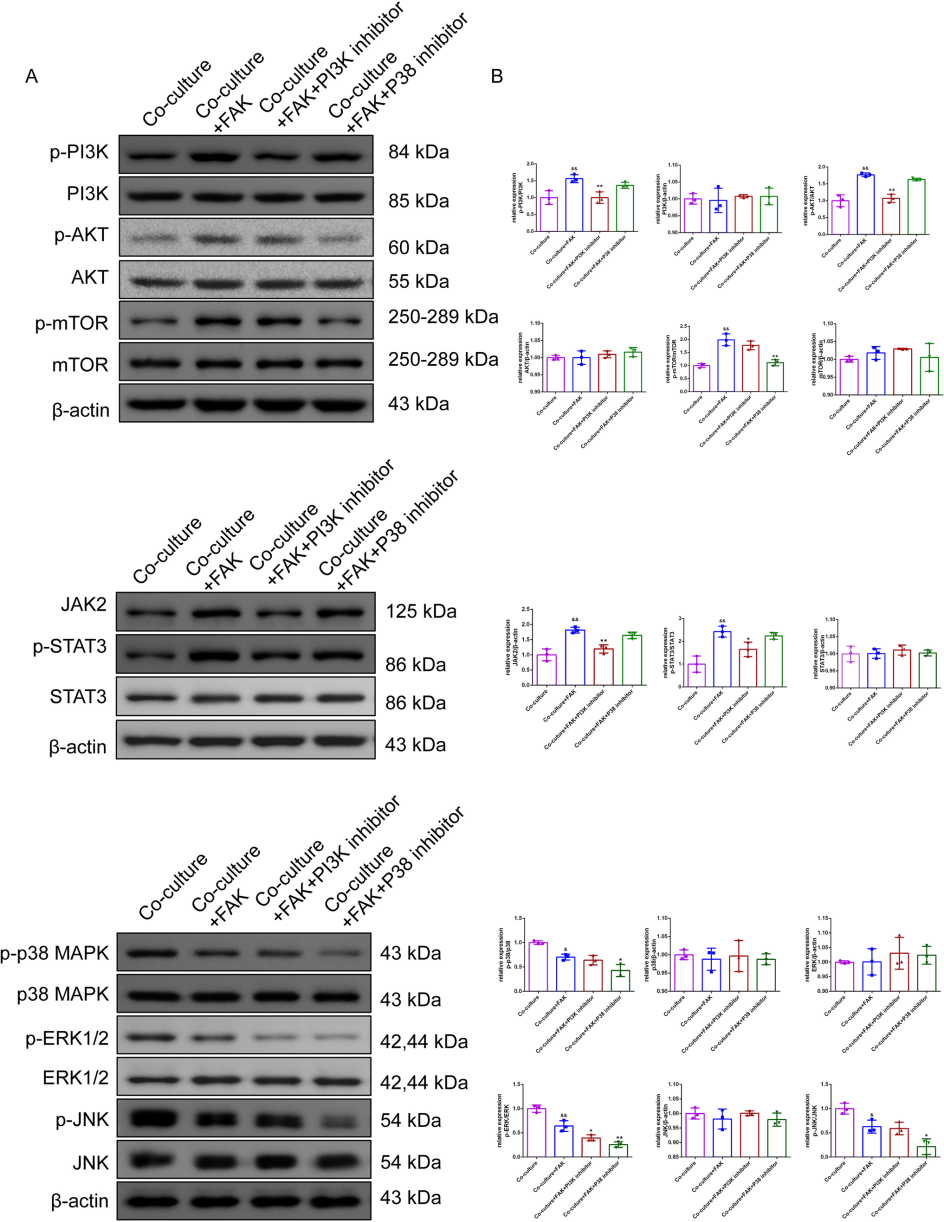
FAK promotes the polarization of macrophages in the Treg-macrophage co-culture system through PI3K/AKT/JAK/STAT3 and p38/JNK/ERK signal pathway. **A, B** The expression of the PI3K, p-PI3K/AKT, p-AKT, JAK2, STAT3, p-STAT3 and p38, p-p38/JNK, p-JNK/ERK1/2, p-ERK1/2 in Treg-macrophage co-culture system after the treatment of FAK (200 μM) and PI3K inhibitor(LY294002) and P38 inhibitor(SB203580) were observed by the Western blot, *n* = 3 in each group, ^&^*P* < 0.05, ^&&^*P* < 0.01 verses co-culture group, ^*^P < 0.05, ^**^P < 0.01 verses co-culture + FAK group. *Note* *PI3K* Phosphatidylinositide 3-kinases; *JAK* Janus Kinase, *STAT3* signal transducer and activator of transcription 3, p38 *MAPK* Mitogen-activated protein kinases, *JNK* Jun N-terminal Kinase

In Figs. 3A, B and 4A, B, Western blot was used to attest the likely signal pathway the FAK involved. The treatment of the FAK and its activator addition, especially the FAK (200 μM) group resulted in the augment of the p-PI3K/p-AKT/p-mTOR/p-FAK/FAK/JAK2/p-STAT3, and the reduction of the p-p38/p-ERK1/2/p-JNK in the macrophages than the control group. The activator of the FAK based on FAK (200 μM) had a more obvious effect relative to the FAK (200 μM) group. The inhibitor FAK based on the FAK (200 μM) demonstrated the opposite way (*P* < 0.05 or *P* < 0.01). In addition, it resulted in an enhancement of the p-PI3K/PI3K, p-AKT/AKT, p-mTOR/mTOR, p-STAT3/STAT3 ratio and JAK2 expression whereas the reduction of the p-p38 MAPK/p38 MAPK, p-ERK/ERK, p-JNK/JNK ratio in the co-culture + FAK than co-culture group (*P* < 0.01). With the addition of the PI3K inhibitor, the p-PI3K/PI3K, p-AKT/AKT, p-STAT3/STAT3, p-ERK/ERK ratio, JAK2 expression was lower than the co-culture + FAK group (*P* < 0.05, *P* < 0.01 respectively). With the addition of the P38 inhibitor, the p-mTOR/mTOR, p-p38 MAPK/p38 MAPK, p-ERK/ERK, p-JNK/JNK ratio was lower than co-culture + FAK group (*P* < 0.05, *P* < 0.01 respectively).

#### FAK promotes cell proliferation, migration, and invasion of HepG2 and SMMC7721 cells processed with the Treg-macrophage co-culture supernatant

**Fig. 5:**
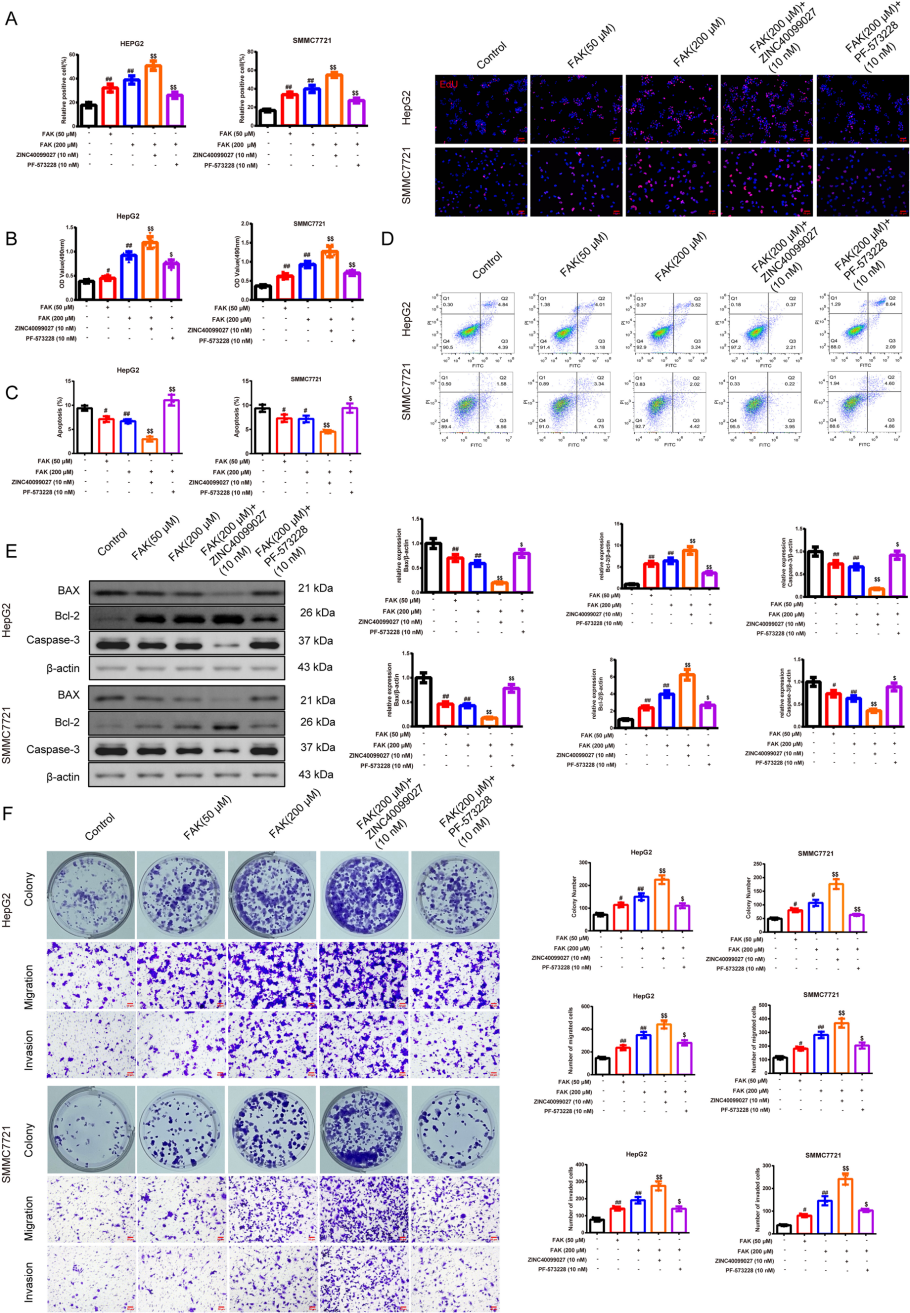
FAK promotes cell proliferation, migration, and invasion of HepG2 and SMMC7721 cells processed with the Treg-macrophage co-culture supernatant. **A, B** EdU (magnification, × 200, scale bar = 50 μm) and MTT were used to test the cell proliferation and vitality after the treatment of the dosages of FAK and its activator ZINC40099027 and inhibitor PF-573228 in HepG2 and SMMC7721 cells,* n* = 3 in each group; **C–E** FCM and western blot were used to evaluate the cell apoptosis in each group, and the results of expression of BAX, Bcl-2, Caspase 3 in each group were represented in graphs. *n* = 3 in each group; **F** Colony formation assay and transwell assay were used to test the ability of cell proliferation, migration, and invasion in HepG2 and SMMC7721 cells, (magnification, × 200, scale bar = 50 μm),* n* = 3 in each group. ^#^*P* < 0.05, ^##^*P* < 0.01 verses control group, ^$^*P* < 0.05, ^$$^*P* < 0.01 verses FAK (200 μM) group. *Note* *EdU* 5-Ethynyl-2′- deoxyuridine, *MTT* 3-(4,5-Dimethylthiazol-2-yl)-2,5-diphenyltetrazolium bromide, *BAX* B-cell lymphoma-2, *Bcl-2* BCL2-Associated X

To clarify whether FAK influenced the HepG2 and SMMC7721 cells processed with the Treg-macrophage co-culture supernatant, we further probed the cell proliferation, cell apoptosis, cell migration, and invasion ability of the HepG2 and SMMC7721 cells. In Fig. 5A–E, results demonstrated that the HepG2 and SMMC7721 cells became active in DNA replication and proliferation whereas they were less active in the apoptosis process after FAK interfered in comparison with the control group, especially in the FAK (200 μM) group. In addition, the FAK activator based on the FAK (200 μM) group had a further effect than the FAK (200 μM) group; the FAK inhibitor addition demonstrated the opposite way (*P* < 0.05). In Fig. 5F, we firmly attested that cell proliferation, migration, and invasion in HepG2 and SMMC7721 cells with FAK had strengthened versus the control group, 200 μM FAK exerted the better influence. The activator and inhibitor of the FAK based on the 200 μM FAK performed the same way as above (*P* < 0.05).

#### FAK promotes epithelial-mesenchymal transition (EMT) in HepG2 and SMMC7721 cells processed with the Treg-macrophage co-culture supernatant

**Fig. 6:**
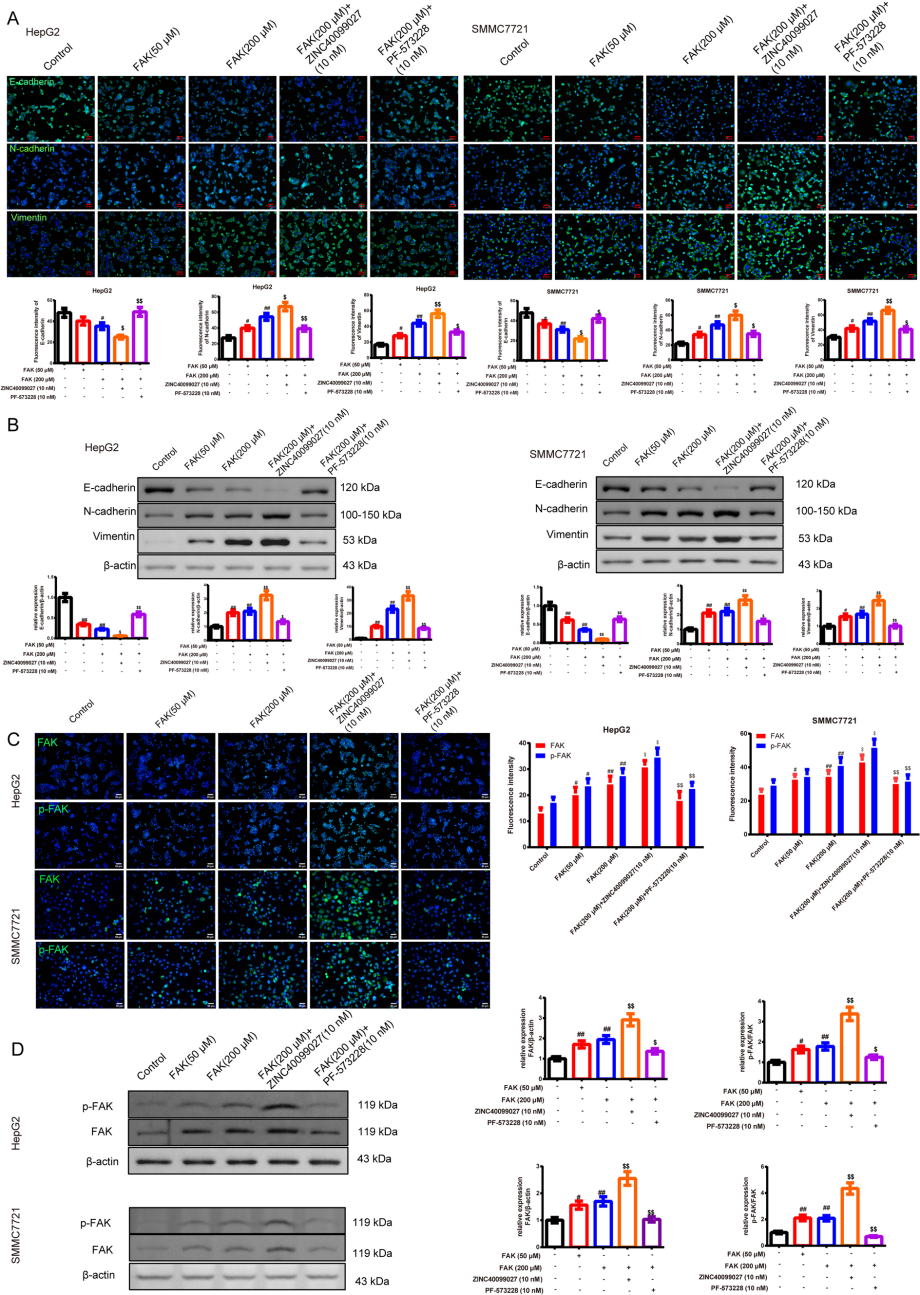
FAK promotes EMT in HepG2 and SMMC7721 cells processed with the Treg-macrophage co-culture supernatant. **A, B** The immunofluorescence and western blot were used to test the E-cadherin, N-cadherin, and Vimentin expression of each group in HepG2 and SMMC7721 cells (magnification, × 200, scale bar = 50 μm) **C, D** The expression of FAK and p-FAK protein in HepG2 and SMMC7721 cells were evaluated by the immunofluorescence and western blot,* n* = 3 in each group; ^#^*P* < 0.05, ^##^*P* < 0.01 verses control group, ^$^*P* < 0.05, ^$$^*P* < 0.01 verses FAK (200 μM) group. *Note* *EMT* Epithelial-mesenchymal transition

In Fig. 6A, B, IF and western blot were used to observe the EMT process. We found that FAK upregulated N-cadherin and Vimentin, downregulated E-cadherin. FAK (200 μM) group exerted a better way to up/down-regulate the EMT-related factors compared to the control group (*P* < 0.05 or *P* < 0.01). Similarly, the FAK activator based on the FAK (200 μM) group had a further effect compared to the FAK (200 μM) group, while The FAK inhibitor addition demonstrated the opposite way (*P* < 0.05). Moreover, in Fig. 6C, D, we further attested that the FAK and FAK activator based on the 200 μM FAK upregulated the expression of the p-FAK and FAK in the HepG2 and SMMC7721 cells (*P* < 0.05 or *P* < 0.01).

#### FAK regulates PI3K/AKT/JAK/STAT3 and p38/JNK signal pathway of HepG2 and SMMC7721 cells processed with the Treg-macrophage co-culture supernatant

**Fig. 7:**
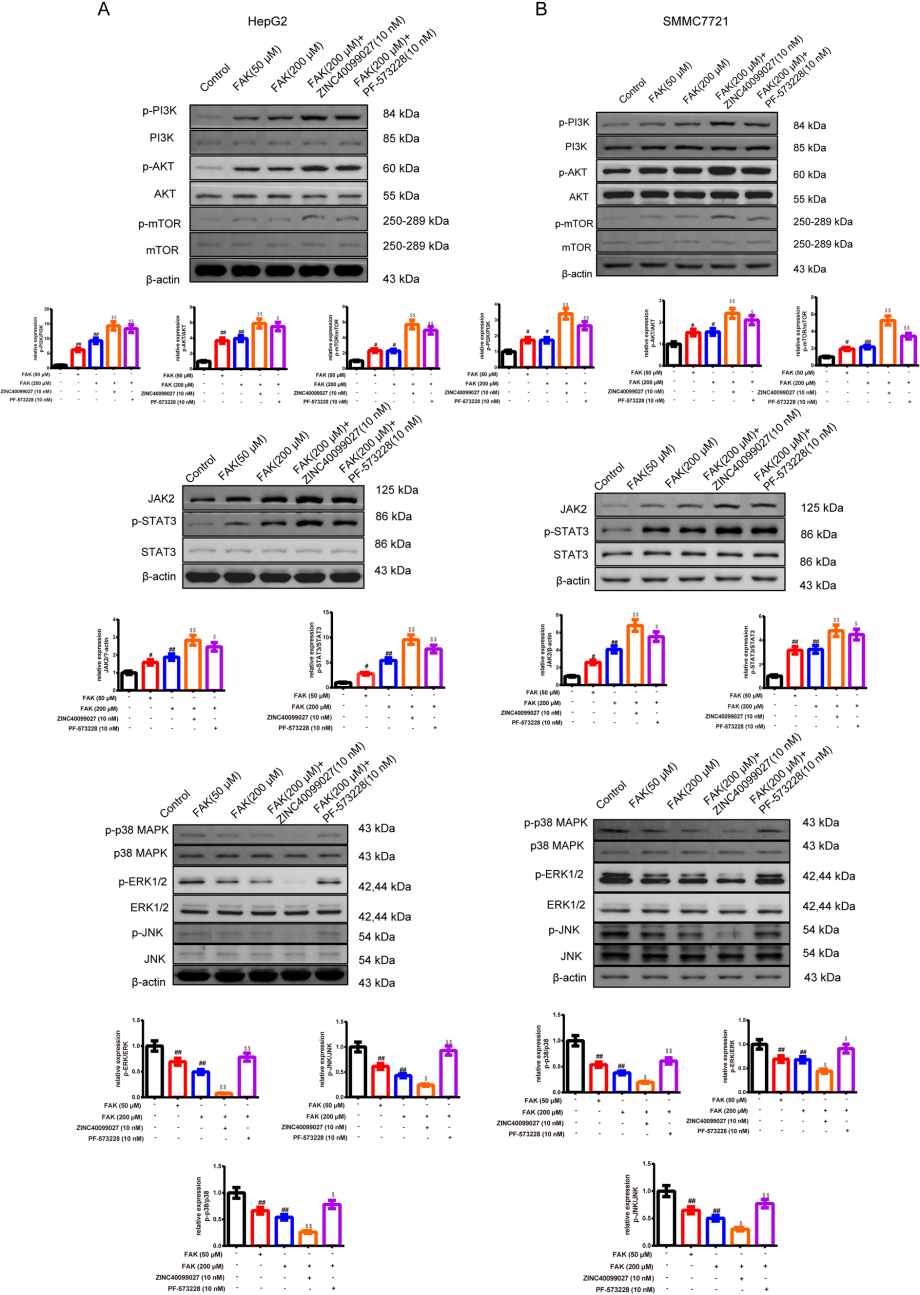
FAK promotes PI3K/AKT, JAK/STAT3 and inhibits p38/JNK/ERK signal pathway of HepG2 and SMMC7721 cells processed with the Treg-macrophage co-culture supernatant. **A, B** The expression of the PI3K, p-PI3K /AKT, p-AKT, JAK2/STAT3, p-STAT3 and p38, p-p38/JNK, p-JNK/ERK1/2, p-ERK1/2 that HepG2 and SMMC7721 cells released after the treatment of the dosages of FAK and its activator and inhibitor were observed by the western blot, *n* = 3 in each group. ^#^*P* < 0.05, ^##^*P* < 0.01 verses control group, ^$^*P* < 0.05, ^$$^*P* < 0.01 verses FAK (200 μM) group

In Fig. 7A, B, FAK and its activator, especially FAK (200 μM) group resulted in the augment of the p-PI3K/p-AKT/p-mTOR/JAK2/p-STAT3, and the reduction of the p-p38/p-ERK1/2/p-JNK in the macrophages in relative to the control group. The addition of the FAK activator had a strengthened effect, while FAK inhibitor addition demonstrated the opposite way than the FAK (200 μM) group in HepG2 and SMMC7721 cells (*P* < 0.05).

### PL exerted an effect on H22 tumor-bearing mouse inhibiting FAK expression and polarization of macrophages

**Fig. 8:**
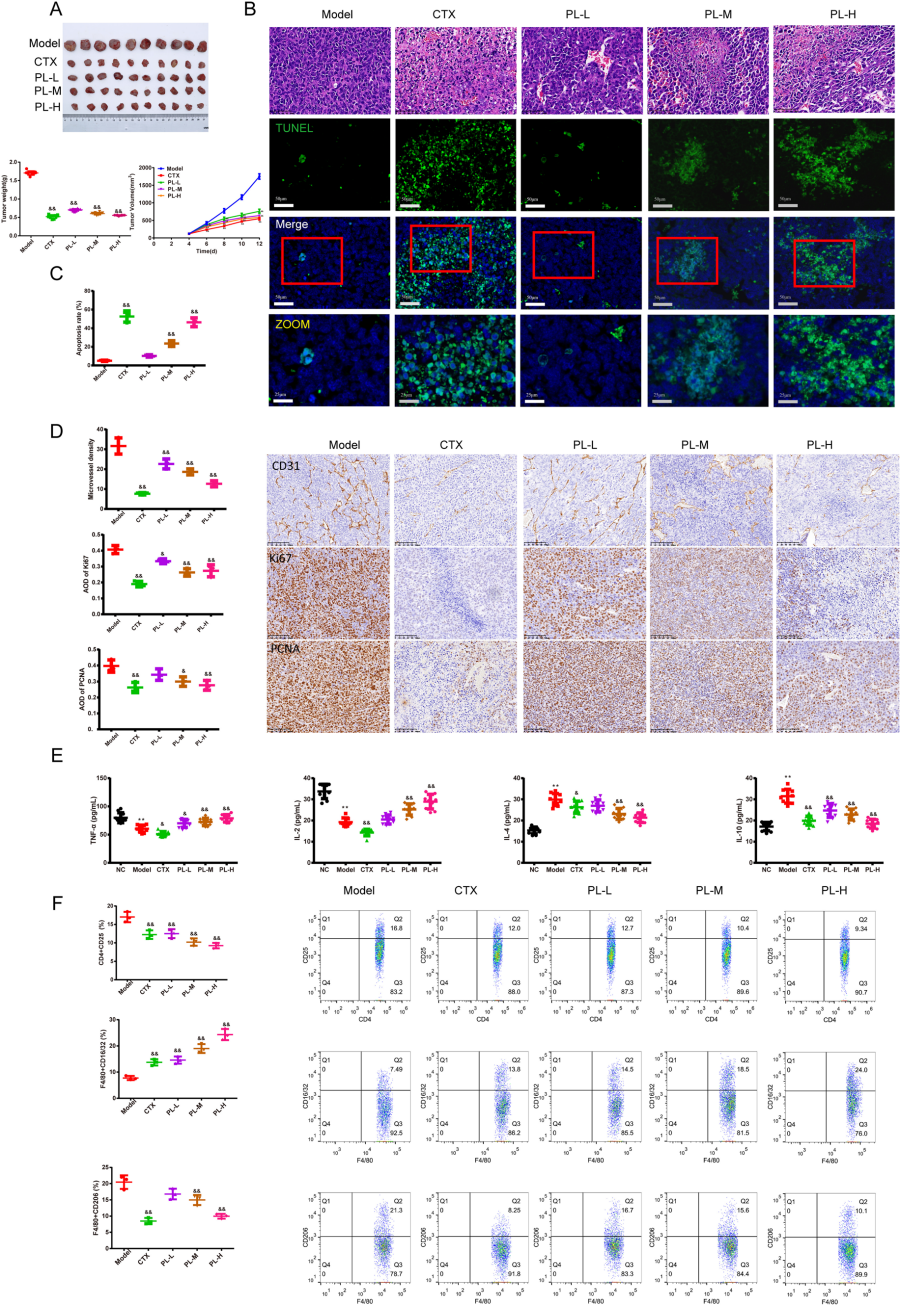
PL exerted an anti-tumor effect on H22 tumor cell-bearing mice. **A** The H22 tumor volume in mice after the treatment of the different dosages of the PL and CTX in each group was recorded in 12 days. And the tumor weight was recorded on the 12th day,* n* = 10 in each group; **B, C** The histomorphology of the H22 tumor tissue in mice was observed by HE staining (magnification × 200, scale bar = 50 μm). TUNEL staining was used to observe the apoptosis of H22 tumor cells in mice, (magnification, × 200, scale bar = 50 μm, ZOOM, × 400, scale bar = 25 μm), and the apoptosis rate was calculated,* n* = 3 in each group; **D** The positive expressions of CD31, Ki67, and PCNA of the H22 tumor tissue in mice were examined by IHC (magnification × 200, scale bar = 50 μm), the average of density was calculated and showed in graphs.* n* = 3 in each group; **E** The TNF-*α*, IL-2, IL-4, and IL-10 contents of H22 tumor cells in mice were observed by the ELISA kits,* n* = 10 in each group. **F** FCM was used to test the percentage of CD25 + CD4 + in Treg cells, F4/80^+^CD16/32^+^ M1, and F4/80^+^CD206^+^M2 macrophages of H22 tumor cells in mice, the results were demonstrated as graphs. *n* = 3 in each group; ^*^P < 0.05, ^**^P < 0.01 verses NC group, ^&^*P* < 0.05, ^&&^*P* < 0.01 verses model group; *Note* *CTX* Cyclophosphamide, *PL* Phellinus linteus, *PL-L* Phellinus linteus with low dosage, *PL-M* Phellinus linteus with medium dosage, *PL-H* Phellinus linteus with high dosage, *PCNA* Proliferating cell nuclear antigen

In Fig. 8A, we found that after the different dosages of the PL or CTX, the tumor volume of the H22 tumor-bearing mice was decreased than the model group within 12 days, and the tumor weight on the 12th day was notably reduced (*P* < 0.01). In Fig. 8B, C, PL and CTX groups had different degrees of vacuoles, and the tumor tissue arrangement was looser than the model group. Apart from that, the percentage of the apoptosis of the tumor cells was increased with the PL or CTX, especially PL with high dosage (*P* < 0.01). The expressions of the CD31, Ki67, and PCNA were less than the model mice (Fig. 8D *P* < 0.05 or *P* < 0.01). In Fig. 8E, the content of the IL-10, and IL-4 in PL and CTX groups were lower than the model group. In contrast, IL-2, and TNF-*α* expressed the opposite way (*P* < 0.01). In Fig. 8F, the percentage of CD4^+^CD25^+^Treg cells and F4/80^+^CD206^+^macrophages were lower whereas the percentage of F4/80^+^CD16/32^+^ macrophages was elevated in the different dosages of the PL and CTX groups than the model group. (*P* < 0.05).

In Fig. S1A, B, with PL and CTX, especially the PL-H group, it resulted in the reduction of the p-PI3K/p-AKT/p-mTOR/p-FAK/FAK/JAK2/p-STAT3 and the increase of the p-p38/p-ERK1/2/p-JNK than the model group (*P* < 0.05 or *P* < 0.01). In Fig. S1C, with the double immunofluorescence, the fluorescence intensity of p-FAK and CD206 both got reduced while the CD16/32 was enhanced with PL in dose-dependent and CTX than model mice (*P* < 0.01).

## Discussion

FAK is a nonreceptor protein tyrosine kinase that is frequently overexpressed in a variety of cancers including HCC [21]. Besides, FAK activation, via autophosphorylation at Tyrosine-397, increases with tumor progression. In addition, it has been reported that integrin-mediated FAK signaling is strongly dependent on integrin endocytosis. This means that the internalization of integrins into endosomes is a critical step for FAK signaling to occur [22]. In this study, we found that in C57BL/6-FAK^−/−^ tumor-bearing mice, FAK promoted the tumor development of HCC including the increased tumor weight and volume. What’s more, FAK resulted in the enhancement of polarization that the more TAM cells develop to the M2 macrophage as well as the increased Treg cells. It has been reported that FAK inhibitors reduced the immunosuppressive cells (TAMs and Treg cells) and consequently impacted effector T cell infiltration into the TME of pancreatic ductal adenocarcinoma KPC mouse models [15]. All these data indicate that inhibiting FAK is a protective way to strengthen in the HCC mice.

Following our initial observations, we conducted in vitro experiments. The results demonstrated that upon stimulation with FAK and its activator, macrophages in the Treg-macrophage co-culture system transitioned from an M1-like phenotype to an M2-like phenotype. In contrast, the FAK inhibitor prompted the opposite effect. M2 macrophages primarily exert their immunosuppressive roles by secreting cytokines such as IL-10 and TGF-*β*. These M2 macrophages hinder the activation of T cells through standard antigen presentation and suppress the immune defense capabilities of T cells, thereby facilitating tumor progression [23]. A previous study declared that M2 macrophage in TAM via the induce of TGF-*β*1 and IL-10 could favor the accumulation of Treg by conversion and chemotaxis [24]. Another study showed that M2 macrophage and Treg cells were positively correlated [25]. Besides, various studies were proving that M2 macrophage via the FAK pathway plays a role in cancer cell metastasis [26–28]. These studies mentioned that M2 macrophage and Treg cells had a close relationship and indicated that not only could the M2 macrophage cause the accumulation of Treg, but also the Treg cells had an impact toward macrophage, making it possible to polarize to the M2-like macrophages, and the vital regulatory factor is proved to be FAK.

What’s more, we applied Treg-macrophage co-culture supernatant in HCC cells (HepG2 and SMMC7721). Results found that after the FAK and its activator addition treatment, it promoted cell proliferation, migration, invasion, and EMT process that was dramatically related to the metastasis of tumor cells, and the FAK inhibitor acted conversely. The study proved that inhibitors of FAK promote tumor cell apoptosis [29]. FAK signaling in cancer-associated fibroblasts promotes breast cancer cell migration and metastasis [30]. Apart from that, activating FAK induces breast cancer cell EMT [31, 32]. In addition, we believed that the Treg-macrophage co-culture supernatant promotes the M2/M1 macrophages. The effect of M2 macrophages and FAK altogether enhances the EMT in HCC. In Jin’s study, they illustrated that inhibiting FAK and re-polarizing TAM promoted the M2-to-M1 phenotype to increase TNF-*α*, but attenuate TGF-*β*, remodeling the TAM and suppressing EMT [33]. Hence, targeting FAK to influence macrophages is a promising method for HCC therapy [34].

As anticipated, our findings demonstrated that PL possesses potent anti-tumor, anti-angiogenic, and immunomodulatory effects in H22 cell-bearing mice, culminating in apoptosis. Previous study mentioned that DBL may act as a potential candidate of the PL to inhibit lung cancer metastasis. It does this by inhibiting the activity of MMP-2 and MMP-9 through affecting various signaling pathways, including PI3K/AKT, MAPKs, FAK/paxillin [16]. In addition, it has been reported that hispolon is an active phenolic compound of PL, a mushroom that has recently been shown to have antioxidant and anticancer activities, exerting an inhibitory effect on the metastasis of SK-Hep1 cells, inhibiting the phosphorylation of FAK [35]. By inhibiting FAK phosphorylation, PL disrupts these pro-metastatic signaling pathways, contributing to its observed antimetastatic effects. We employed computational modeling to predict the binding mode and affinity of PL to FAK to find that the compositions of PL (DBL, hispolon) had high affinity with FAK. In line with our results, Jeong's research reinforces the synergistic anti-tumor potential of PL when combined with radiation, underscoring the scientific basis for considering PL as a radiosensitizer in HCC [36]. To elucidate the mechanisms through which PL influences HCC, we examined FAK expression in conjunction with Treg and macrophage cells. Our findings indicated that FAK expression inversely correlated with PL, which resulted in reduced Treg cells and a decreased M2/M1 macrophage ratio. Thus, we postulate that PL suppresses FAK expression, leading to a reduction in Treg cells and subsequently promoting a shift in macrophage polarization from M2 to M1.

Studies proved that activated FAK phosphorylates the downstream PI3K and then activates AKT [37]. To further elucidate the molecular signaling pathway FAK involved, we tested the downstream PI3K/AKT/mTOR, JAK/STAT3, and p38/JNK signaling. In malignant glioblastoma cell lines, the levels of phosphorylated JAK1, JAK2, and STAT3 were uniformly upregulated with FAK and positively correlated, followed by the levels of phosphorylated p38, JNK and ERK1/2 downregulated, and the trend was reversed after treatment [38]. A similar study reported that FAK/PI3K/AKT signaling mediates the HCC [39]. Consistently, in our study, we proved that PL resulted in the reduction of the p-PI3K/p-AKT/p-mTOR/JAK2/p-STAT3 and the augment of the p-p38/p-ERK1/2/p-JNK in the H22 tumor-bearing mice.

In this study, we discerned that FAK plays a pivotal role in HCC metastasis. The inhibition of both FAK and PL notably induced a shift in macrophage polarization from M2 to M1 and reduced Treg cells, thereby enhancing immune function. Regarding the compositions of PL, we have now incorporated a detailed discussion on their various types and roles, particularly in the context of our study's focus. While our current research does not dissect the individual compositions of PL, we recognize the importance of this aspect. As such, we will explore the detailed compositions of PL and their functional implications. We believe that this will significantly contribute to the field and address a critical gap in the current understanding.

To sum up, the present study indicated that FAK promoted HCC through increasing Treg cells to polarize macrophages from M1 to M2 via PI3K/AKT/JAK/STAT3, and p38/JNK pathway. Moreover, PL demonstrated potential therapeutic benefits against HCC. These findings offer a novel avenue for future clinical and experimental investigations into HCC.

### Supplementary Information

Below is the link to the electronic supplementary material.

Supplementary file1 (DOCX 1441 KB)

## Data Availability

The datasets generated during and/or analyzed during the current study are available from the corresponding author upon reasonable request.

## References

[1] Vogel A, Meyer T, Sapisochin G, Salem R, Saborowski A (2022). Hepatocellular carcinoma. Lancet (London, England).

[2] Zou H, Li M, Lei Q, Luo Z, Xue Y, Yao D (2022). Economic burden and quality of life of hepatocellular carcinoma in greater China: a systematic review. Front Public Health.

[3] Pelosof L, Lemery S, Casak S, Jiang X, Rodriguez L, Pierre V (2018). Benefit-risk summary of regorafenib for the treatment of patients with advanced hepatocellular carcinoma that has progressed on sorafenib. Oncologist.

[4] Golden-Mason L, Rosen HR (2017). Galectin-9: diverse roles in hepatic immune homeostasis and inflammation. Hepatology (Baltimore, MD).

[5] Murray PJ (2017). Macrophage polarization. Annu Rev Physiol.

[6] Zhu Z, Zhang H, Chen B, Liu X, Zhang S, Zong Z (2020). PD-L1-mediated immunosuppression in glioblastoma is associated with the infiltration and M2-polarization of tumor-associated macrophages. Front Immunol.

[7] Granito A, Muratori L, Lalanne C, Quarneti C, Ferri S, Guidi M (2021). Hepatocellular carcinoma in viral and autoimmune liver diseases: role of CD4+ CD25+ Foxp3+ regulatory T cells in the immune microenvironment. World J Gastroenterol.

[8] Flecken T, Schmidt N, Hild S, Gostick E, Drognitz O, Zeiser R (2014). Immunodominance and functional alterations of tumor-associated antigen-specific CD8+ T-cell responses in hepatocellular carcinoma. Hepatology (Baltimore, MD).

[9] Trehanpati N, Vyas AK (2017). Immune regulation by T regulatory cells in hepatitis B virus-related inflammation and cancer. Scand J Immunol.

[10] Xie Y, Zhang Y, Wei X, Zhou C, Huang Y, Zhu X (2020). Jianpi Huayu decoction attenuates the immunosuppressive status of H(22) hepatocellular carcinoma-bearing mice: by targeting myeloid-derived suppressor cells. Front Pharmacol.

[11] Zhou SL, Zhou ZJ, Hu ZQ, Huang XW, Wang Z, Chen EB (2016). Tumor-associated neutrophils recruit macrophages and T-regulatory cells to promote progression of hepatocellular carcinoma and resistance to sorafenib. Gastroenterology.

[12] Zhuo Q, Yu B, Zhou J, Zhang J, Zhang R, Xie J (2019). Lysates of *Lactobacillus acidophilus* combined with CTLA-4-blocking antibodies enhance antitumor immunity in a mouse colon cancer model. Sci Rep.

[13] Lee BY, Timpson P, Horvath LG, Daly RJ (2015). FAK signaling in human cancer as a target for therapeutics. Pharmacol Ther.

[14] Serrels A, Lund T, Serrels B, Byron A, McPherson RC, von Kriegsheim A (2015). Nuclear FAK controls chemokine transcription, Tregs, and evasion of anti-tumor immunity. Cell.

[15] Jiang H, Hegde S, Knolhoff BL, Zhu Y, Herndon JM, Meyer MA (2016). Targeting focal adhesion kinase renders pancreatic cancers responsive to checkpoint immunotherapy. Nat Med.

[16] Chao W, Deng JS, Li PY, Liang YC, Huang GJ (2017). 3,4-Dihydroxybenzalactone suppresses human non-small cell lung carcinoma cells metastasis via suppression of epithelial to mesenchymal transition, ROS-mediated PI3K/AKT/MAPK/MMP and NFκB signaling pathways. Molecules (Basel, Switzerland).

[17] Kim HM, Kang JS, Kim JY, Park SK, Kim HS, Lee YJ (2010). Evaluation of antidiabetic activity of polysaccharide isolated from Phellinus linteus in non-obese diabetic mouse. Int Immunopharmacol.

[18] Fangbin lv N, yu S (2015). Research progress on anti-tumor mechanism of Phellinus pullus. Shanghai J Tradit Chin Med.

[19] Seo JH, Sung YH, Kim KJ, Shin MS, Lee EK, Kim CJ (2011). Effects of Phellinus linteus administration on serotonin synthesis in the brain and expression of monocarboxylate transporters in the muscle during exhaustive exercise in rats. J Nutr Sci Vitaminol.

[20] Sasaki S, Nishikawa J, Sakai K, Iizasa H, Yoshiyama H, Yanagihara M (2019). EBV-associated gastric cancer evades T-cell immunity by PD-1/PD-L1 interactions. Gastric Cancer Off J Int Gastric Cancer Assoc Jpn Gastric Cancer Assoc.

[21] Osipov A, Saung MT, Zheng L, Murphy AG (2019). Small molecule immunomodulation: the tumor microenvironment and overcoming immune escape. J Immunother Cancer.

[22] Alanko J, Ivaska J (2016). Endosomes: emerging platforms for integrin-mediated FAK signalling. Trends Cell Biol.

[23] Li J, Kim SY, Lainez NM, Coss D, Nair MG (2021). Macrophage-regulatory T cell interactions promote type 2 immune homeostasis through resistin-like molecule α. Front Immunol.

[24] Wang J, Huang H, Lu J, Bi P, Wang F, Liu X (2017). Tumor cells induced-M2 macrophage favors accumulation of Treg in nasopharyngeal carcinoma. Int J Clin Exp Pathol.

[25] Aliyah SH, Ardiyan YN, Mardhiyah I, Herdini C, Dwianingsih EK, Aning S (2021). The distribution of M2 macrophage and Treg in nasopharyngeal carcinoma tumor tissue and the correlation with TNM status and clinical stage. Asian Pac J Cancer Prev: APJCP.

[26] Sun Y, Qian Y, Chen C, Wang H, Zhou X, Zhai W (2022). Extracellular vesicle IL-32 promotes the M2 macrophage polarization and metastasis of esophageal squamous cell carcinoma via FAK/STAT3 pathway. J Exp Clin Cancer Res.

[27] Ngabire D, Niyonizigiye I, Patil MP, Seong YA, Seo YB, Kim GD (2020). M2 macrophages mediate the resistance of gastric adenocarcinoma cells to 5-fluorouracil through the expression of integrin β3, focal adhesion kinase, and cofilin. J Immunol Res.

[28] Wu J, Wang Y, Yang Y, Liu F, Jiang Z, Jiang Z (2022). TNFSF9 promotes metastasis of pancreatic cancer by regulating M2 polarization of macrophages through Src/FAK/p-Akt/IL-1β signaling. Int Immunopharmacol.

[29] Mustafa M, Abd El-Hafeez AA, Abdelhamid D, Katkar GD, Mostafa YA, Ghosh P (2021). A first-in-class anticancer dual HDAC2/FAK inhibitors bearing hydroxamates/benzamides capped by pyridinyl-1,2,4-triazoles. Eur J Med Chem.

[30] Wu HJ, Hao M, Yeo SK, Guan JL (2020). FAK signaling in cancer-associated fibroblasts promotes breast cancer cell migration and metastasis by exosomal miRNAs-mediated intercellular communication. Oncogene.

[31] Luo J, Yao JF, Deng XF, Zheng XD, Jia M, Wang YQ (2018). 14, 15-EET induces breast cancer cell EMT and cisplatin resistance by up-regulating integrin αvβ3 and activating FAK/PI3K/AKT signaling. J Exp Clin Cancer Res.

[32] Chen J, Zhao D, Zhang L, Zhang J, Xiao Y, Wu Q (2022). Tumor-associated macrophage (TAM)-derived CCL22 induces FAK addiction in esophageal squamous cell carcinoma (ESCC). Cell Mol Immunol.

[33] Jin H, He Y, Zhao P, Hu Y, Tao J, Chen J (2019). Targeting lipid metabolism to overcome EMT-associated drug resistance via integrin β3/FAK pathway and tumor-associated macrophage repolarization using legumain-activatable delivery. Theranostics.

[34] Zheng X, Turkowski K, Mora J, Brüne B, Seeger W, Weigert A (2017). Redirecting tumor-associated macrophages to become tumoricidal effectors as a novel strategy for cancer therapy. Oncotarget.

[35] Huang GJ, Yang CM, Chang YS, Amagaya S, Wang HC, Hou WC (2010). Hispolon suppresses SK-Hep1 human hepatoma cell metastasis by inhibiting matrix metalloproteinase-2/9 and urokinase-plasminogen activator through the PI3K/Akt and ERK signaling pathways. J Agric Food Chem.

[36] Jeong YK, Oh JY, Yoo JK, Lim SH, Kim EH (2020). The biofunctional effects of mesima as a radiosensitizer for hepatocellular carcinoma. Int J Mol Sci.

[37] Oudart JB, Doué M, Vautrin A, Brassart B, Sellier C, Dupont-Deshorgue A (2016). The anti-tumor NC1 domain of collagen XIX inhibits the FAK/ PI3K/Akt/mTOR signaling pathway through αvβ3 integrin interaction. Oncotarget.

[38] Swiatek-Machado K, Mieczkowski J, Ellert-Miklaszewska A, Swierk P, Fokt I, Szymanski S (2012). Novel small molecular inhibitors disrupt the JAK/STAT3 and FAK signaling pathways and exhibit a potent antitumor activity in glioma cells. Cancer Biol Ther.

[39] Zhang PF, Li KS, Shen YH, Gao PT, Dong ZR, Cai JB (2016). Galectin-1 induces hepatocellular carcinoma EMT and sorafenib resistance by activating FAK/PI3K/AKT signaling. Cell Death Dis.

