# Supplementary material for: Phellinus linteus activates Treg cells via FAK to promote M2 macrophage polarization in hepatocellular carcinoma
Source: Cancer Immunol Immunother. 2024 Jan 19;73(1):18. doi: 10.1007/s00262-023-03592-3 (PMC10799134; doi:10.1007/s00262-023-03592-3)
Supplement: Supplementary file 1 — Supplementary file1 (DOCX 1441 KB) [file 262_2023_3592_MOESM1_ESM.docx]

**
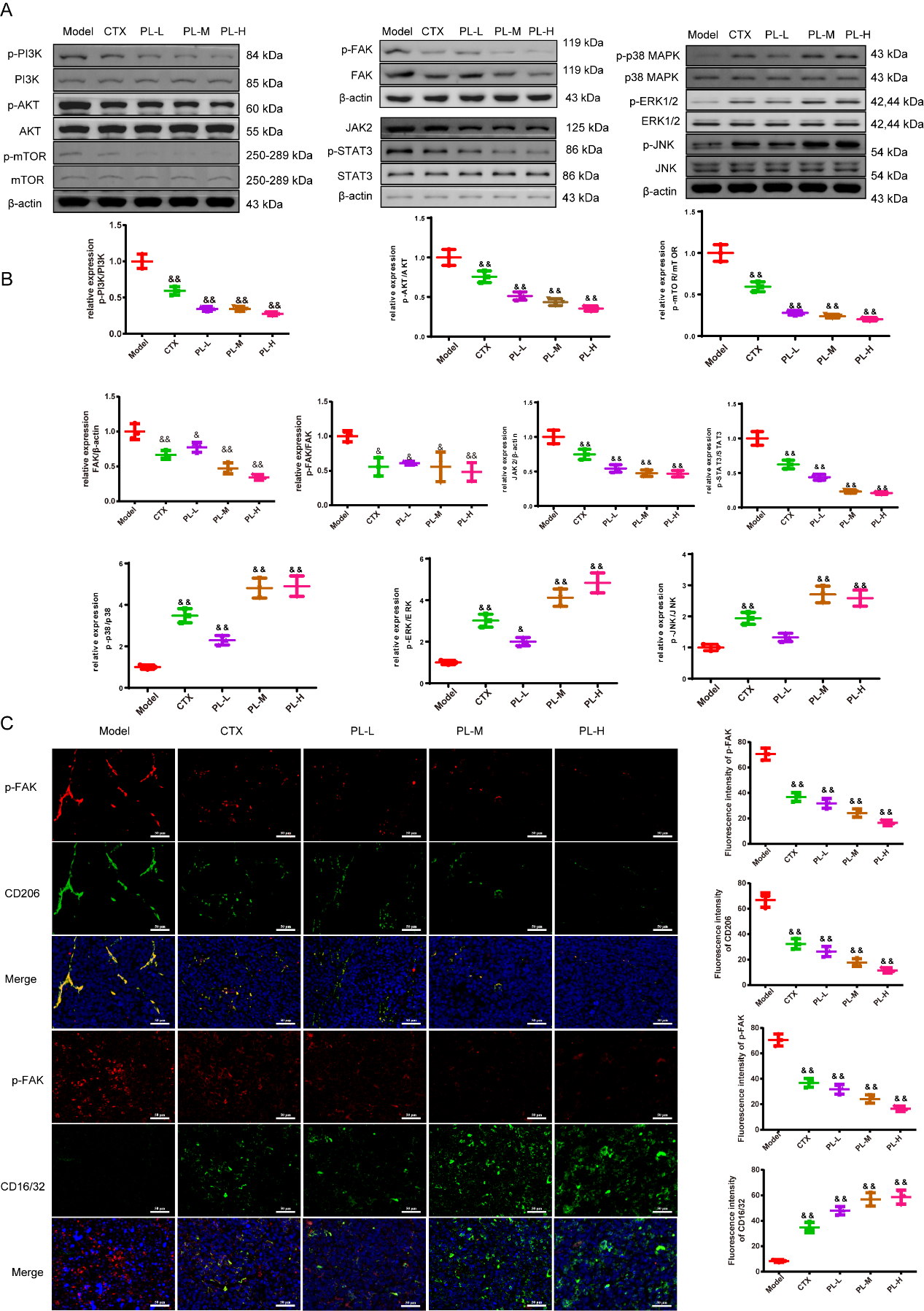
**

**Fig S1** PL inhibited FAK expression and polarization of the macrophages through PI3K/AKT, JAK/STAT3, and p38/JNK signal pathway in H22 tumor-bearing mice

**A-B.** The expression of the PI3K, p-PI3K, AKT, p- AKT, p-FAK, FAK, JAK2/STAT3, p-STAT3 and p38, p-p38/JNK, p-JNK/ERK1/2, p-ERK1/2 of H22 tumor cells in mice after the treatment of the different dosages of the PL and CTX in each group were observed by the western blot, *n*=3 in each group. **C.** Double staining of the immunofluorescence was used to test the p-FAK/CD16 and p-FAK/CD206 protein expression in H22 tumor cells (magnification × 200, scale bar =50 μm), and the immunofluorescence of the p-FAK/CD16/CD206 was calculated respectively, *n*=3 in each group. ^&^*P*<0.05, ^&&^*P*<0.01 vs. model group. **Note:** CTX: Cyclophosphamide; PL-L: Phellinus linteus with low dosage; PL-M: Phellinus linteus with medium dosage; PL-H: Phellinus linteus with high dosage.
